# Prevalence and molecular detection of *Staphylococcus aureus* resistance to antibiotics

**DOI:** 10.1097/MD.0000000000038562

**Published:** 2024-06-14

**Authors:** Fatima Zahra Dendi, Rachida Allem, Mohammed Sebaihia, Sidahmed Bensefia, Mohammed Cheurfa, Hannan Alamir, Emmanuel Ifeanyi Obeagu

**Affiliations:** a Laboratory of Molecular Biology, Genomics and Bioinformatics, University Hassiba Benbouali of Chlef, Algeria; b Bioresource Laboratory, Faculty of Science of Nature and Live, University Hassiba Benbouali of Chlef, Algeria; c Food Bacteriology laboratory, Pasteur Institute, Dalybrahim, Algiers; d University of Djilali BounaalaKhmismilia, Ain defla, Algiers; e Department of Medical Laboratory Science, Kampala International University, Uganda.

**Keywords:** methicillin-resistant, PCR multiplex, *Staplylococcus aureus*

## Abstract

In Algeria, the issue of antibiotic resistance is on the rise, being the *Staphylococcus aureus* infection as a significant concern of hospital-acquired infections. The emergence of antibiotic resistance in this bacterium poses a worldwide challenge. The aim of this study aims to establish the incidence of *S aureus* strains in Algeria as well as identify phenotypic and genotypic resistance based on the “mecA” and “nuc” genes. From 2014 to 2017, a total of 185 *S aureus* strains were isolated from patients at a hospital in the city of Rouïba, Algiers the number of isolates was slightly higher in males at 58.06% compared to females at 41.94%, resulting in a sex ratio of 1.38. the Oxacillin and Cefoxitin DD test (1 μg oxacillin disk and 30 μg cefoxitin disk) identified 42 strains as resistant. The results indicated high resistance to lactam antibiotics, with penicillin having a 100% resistance rate. There was also significant resistance to oxacillin (51.25%) and cefoxitin (50%). This resistance was frequently associated with resistance to other antibiotic classes, such as aminoglycosides (50%) and Macrolides (28.29%). To confirm methicillin-resistant characteristics, a polymerase chain reaction (PCR) multiplex was conducted on 10 isolates (6 SARM; 4 MSSA) on a phenotypic level. Three isolates tested positive for “mecA,” while 7 were negative. All strains carry the nuc gene, which is specific to *S aureus.* In Algeria, the incidence of *S aureus* resistance is slightly lower compared to other countries, but it is increasing over time. It is now more crucial than ever to restrict the proliferation of multidrug-resistant strains and reduce undue antibiotic prescriptions. To achieve this, it is vital to keep updated on the epidemiology of this bacterium and its antibiotic susceptibility. This will enable the formulation of appropriate preventive control measures to manage its progression.

## 1. Introduction

*Staphylococcus aureus* is a Gram-positive bacterium that can cause a wide range of infections in humans, ranging from mild skin infections to severe life-threatening diseases such as pneumonia and sepsis. The emergence of antibiotic resistance in *S aureus*, particularly methicillin-resistant *S aureus* (MRSA), has become a major public health concern. The prevalence of antibiotic resistance in *S aureus* is influenced by factors such as indiscriminate use of antibiotics, inadequate infection control practices, and the ability of the bacterium to acquire and disseminate resistance genes. MRSA, in particular, has shown resistance not only to beta-lactam antibiotics (such as methicillin, oxacillin, and penicillin) but also to multiple other classes of antibiotics, making treatment challenging. Studies on the prevalence of *S aureus* antibiotic resistance often involve the collection of clinical isolates from patients with various infections. These isolates are then tested against a panel of antibiotics to determine their susceptibility or resistance patterns. Surveillance programs help monitor the changing landscape of antibiotic resistance, providing valuable information for healthcare practitioners and policymakers. In Africa, the prevalence of *S aureus* resistant to oxacilline in Bernini was approximately 36% in 2006, but it declined in 2008 (14,8%). Meanwhile, in Algeria, the MRSA rate has been on a continuous rise, increasing from 4.5% in 2002 to 33.2% in 2004.^[1]^

Molecular techniques play a crucial role in the detection of antibiotic resistance in *S aureus*. polymerase chain reaction (PCR) and other molecular methods are commonly employed to identify specific resistance genes or mutations associated with antibiotic resistance. Some of the key molecular targets for detecting *S aureus* resistance include the mecA gene, which is associated with methicillin resistance, and other genes linked to resistance against different antibiotic classes. Whole-genome sequencing (WGS) has also become a powerful tool for understanding the genomic diversity of *S aureus* strains and their antibiotic resistance profiles. WGS allows researchers to analyze the entire genome of bacterial isolates, identifying genetic variations that contribute to antibiotic resistance. The increasing prevalence of antibiotic-resistant *S aureus* poses significant challenges for healthcare systems worldwide. Limited treatment options for infections caused by these resistant strains can lead to prolonged illnesses, increased healthcare costs, and higher mortality rates. Efforts to combat antibiotic resistance involve the development of new antimicrobial agents, improved diagnostics, and enhanced infection control measures. Additionally, promoting judicious use of antibiotics and implementing surveillance programs are essential components of a comprehensive strategy to mitigate the further spread of antibiotic-resistant *S aureus*. Nowadays, the treatment of bacterial infections is carried out mainly by the use of antibiotic substances which often causes therapeutic impasses. MRSA is considered to be one of the leading causes of morbidity in hospitals; it is ranked second among these infections after *Escherichia coli*.^[2]^

Thus, an epidemiological study is necessary to determine its frequency in hospital settings and to evaluate its resistance profile in order to define some strategy of therapeutic and preventive control. This situation led us to study the current state of this micro-organism on everything that in Algeria little study has been carried out concerning this pathogen.

## 2. Materials and methods

The study was prospective and carried out on patients who contracted staphylococcal infections at the University Hospital of Rouiba, Algeria during the period from 2014 to 2017.

### 2.1. Data collection

Demographic data (age and sex) and clinical information of patients positive for staphylococcal infections were obtained from the Bacteriology Laboratory Records Center.

### 2.2. Bacterial identification

The identification of *S aureus* isolates was carried out using conventional techniques. The morpho-tinctorial evaluation of colonies was carried out by Gram staining, isolation and purification in médium Chapman and biochemical analysis using catalase, coagulase tests, Pastorex Staph plus agglutination, Protein A and identification by Api20Staph. The identified strains were stored on storage agar at −4°C. The positive control used in the study was *S aureus* ATCC 25923. Table 1 shows the origin of *S aureus* strains used in the molecular study

**Table 1:** The origin of *Staphylococcus aureus* strains used in the molecular study.

The strain No.	Type of samples	Sex	Service
01	Pus	♀ Female	MF
02	Pleural Liquid	♂ Male	PN
03	Pus	♂ Male	MS
04	Pus	♂ Male	MS
05	LP	♀ Female	PN
06	Hemoculture	♀ Female	FS
07	Pus	♂ Male	MM
08	Pus	♂ Male	MM
09	Hemoculture	♀ Female	FS
10	Pus	♂ Male	MM

FS/MS: Female Surgery/Male Surgery, LP: Pleural liquid, PN: Pneumology, MF: Medicine Female, MH: Medicine Male.

### 2.3. Sensitivity to antibiotics

The antimicrobial resistance study was conducted following Muller Hilton MH conventional diffusion method according to the recommendations of the National Committee for Clinical Laboratory Standards (NCCLS), Pennsylvania, USA, NCCLS.^[3]^ The disk diffusion test of oxacillin and cefoxitin DD test (oxacillin 1 µg disk, cefoxitin 30 µg disk) is performed to detect the resistance of the isolates. The interpretation of the resistance according to the inhibition diameter of a sensitive *S aureus* oxacillin disc ≥ 13 mm, resistant ≤ 10 mm and a cefoxitin *S aureus* disc sensitive ≥ 22 mm, resistant ≤ 19 mm. Antibiogram of the French Society of Microbiology, Communiquè 2007. In frantibiotic quality control was performed using *S aureus* ATCC 25923 (oxacillin-sensitive strain: MSSA)/*Staplylococcus aureus* ATCC 43300 (oxacillin-resistant strain: MRSA).

### 2.4. Molecular detection of antimicrobial resistance genes

#### 2.4.1. Genomic DNA extraction and purification

The extraction and purification of genomic DNA from young colonies (18–24 hours) were performed using the INVITROGEN PureLINK® Genomic DNA Mini Kit following the manufacturer recommendations. The colonies under investigation were scraped and added to conical tubes containing 1 mL of TE buffer added using a Pasteur pipette. The tubes were centrifuged for 10 minutes and the pellet collected, while the supernatant was discarded. Technical term abbreviations used would be explained upon first use. 18 microliters of genomic digestion buffer were added and vortexed for 10 minutes. Then, 20 microliters of Proteink and 200 microliters of genomic lysis buffer, along with Votrexer, were incubated for 1 hour. Next, 200 microliters of absolute ethanol were added and vortexed.

The resultant solution was transferred to columns and centrifuged at 10,000 rpm for 1 minute. After that, 500 microliters of Wash Buffer 1 were added to the columns and centrifuged at 10,000 rpm for another minute. The containers were discarded, resuspended, and 500 microliters of Wash Buffer were added. Centrifuge the sample at 15,000 revolutions per minute for 3 minutes. Centrifuge the elution again at 15,000 revolutions per minute for 1 minute. Discard the collector and transfer the sample to 1.5 mL tubes on the column. Add 50 µL of genomic elution buffer to the column and centrifuge again at 15,000 revolutions per minute for 1 minute. Discard the column and add another 50 µL of genomic elution buffer. Store the DNA at + 4°C (Genomic DNA extraction was performed as per the manufacturer protocol with no modification).

### 2.5. Molecular detection of “nuc” and “mecA” genes by multiplex PCR

Molecular identification of *S aureus* strains was performed by using *S aureus*-specific “nuc” primers and “mecA” primers responsible for detecting oxacillin resistance. The amplification reaction for the 2 *S aureus* genes was carried out in a total volume of 25 μL containing 5 μL of chromosomal DNA, 0.25 μL of each primer (130 μg/mLeach), 0.25 μL deoxynucleotide triphosphate Mix (25 μM each), 2.5 μL of buffer (10 μL), 1 μL of Mgcl 2 (25 μm), 0.25 μL of Taq polymerase. The volume is supplemented with 15.75μL ultra-pure H_2_O. The amplification conditions included initial denaturation at 94°C for 5 minutes followed by denaturation cycle (30 seconds at 94°C), hybridization (30 seconds at 55°C), (extension at 72°C) and final extension (10 minutes at 72°C). The amplifying fragments were separated by electrophoresis on 1% agarose gel in 1X TBE supplemented with ethidium bromide (0,5 mg) for 45 minutes. The primers used in this study are shown in Table 2. For the 16S analysis, we used the universal primers 27F and 1492R.

**Table 2:** Properties of the pair of primers used for the detection of the genes “mecA,” “nuc” and “ARN16s.”

Primer	Sequence 3’ - 5’	Fragment size
Primer mecA-1	5’ -GGGATCATAGCGTCATTATTC-3’	527 bp
Primer mecA-2:	5’ -AACGATTGTGACACGATAGCC-3’	
Primer nuc-1:	5’-TCAGCAAATGCATCACAAACAG-3’	255 pb
Primer nuc-2:	5’-CGTAAATGCACTTGCTTCAGG-3’	
Primer 16S 27F	5’-AGAGTTTGATCCTGGCTCAG-3’	1500 pb
Primer 16S 1492R	5’-GGTTACCTTGTTACGACTT-3’	

### 2.6. Ethical considerations

Ethical approval was obtained from research ethics committee of Hospital Rouiba, Rue Larbi Abddelsalem, Rouiba, 16000, Alger, Algeria and consents obtained from the patients from been recruited for the study. The choice of the ethical approval body was chosen as the patients were recruited from the same hospital and was the best body to grant access to the patients.

### 2.7. Statistical analysis

Data processing was performed using static software: EXCEL and SPSS statistics version 21.

## 3. Results

The isolation frequency of *S aureus* was variable according to the services, there were 89 cases at the level of resuscitation services, 48.10%. 37 and 35 cases at the level of medical services (surgery and internal medicine). The isolation frequency of *S aureus* depends on the nature of the sample 42.31% was recorded in the pus samples. 26.61% in Hemoculture and 18.95% in ponction (Table 3).

**Table 3:** The frequency of *Staphylococcus aureus* according to the nature of the simples.

Type of sample	Number of isolated staphylococci	Number of *S aureus*	Percentage %
Hemoculture	132	50	26.61%
Pus	209	91	42.31%
Ponction	94	28	18.95%
Drain	71	16	14.31%
Total	496	185	100%

**Figure 1.**
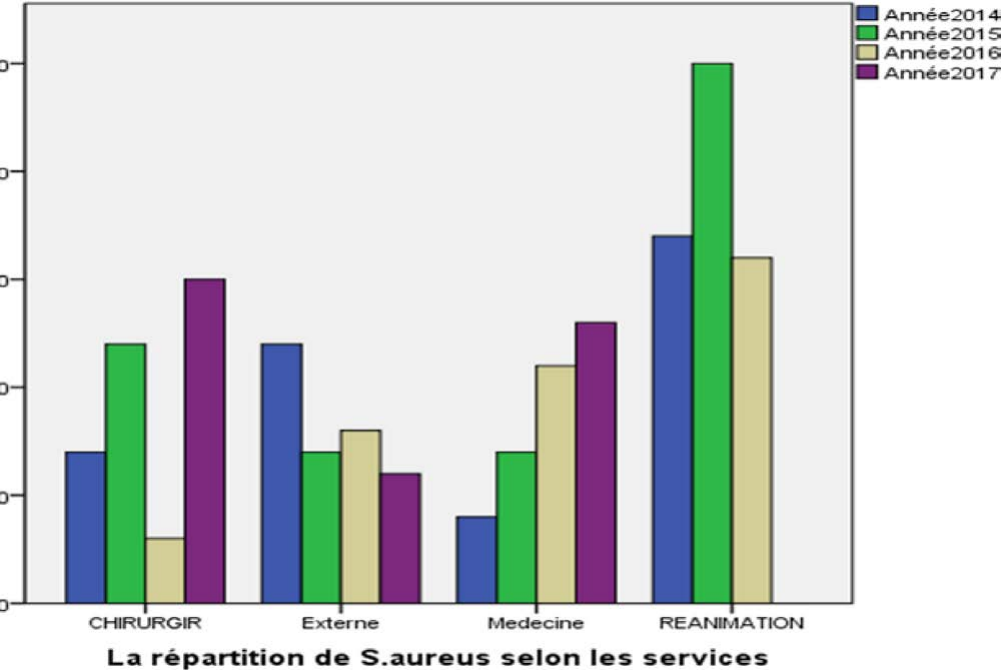
The frequency of Staphylococcus aureus resistance to antibiotics.

**Figure 2.**
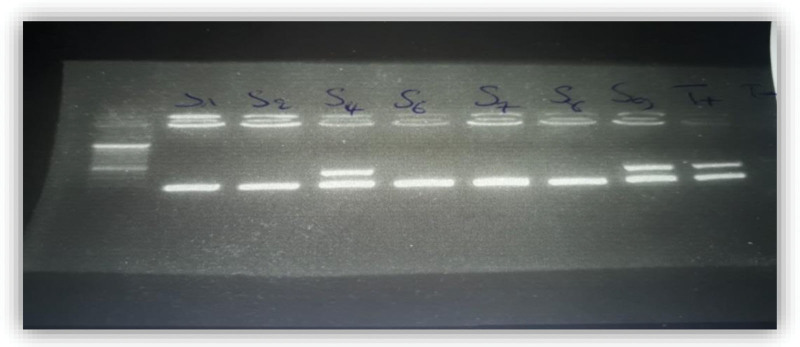
Agarose gel electrophores 1% of the multiplex PCR amplification products of the “mecA” and “nuc” genes, from S1 to S7 of the strains tested.

**Figure 3.**
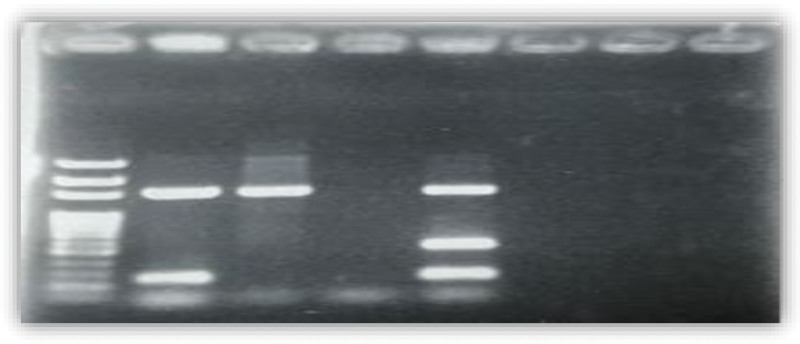
Agarose gel electrophores 1% of the multiplex PCR amplification products of the “mecA” and “nuc” genes, from S8 to S10 of the strains tested (SmartLader 200bp, Eurogentec).

All strains of *S aureus* were tested for antibiotic susceptibility to a range of antibiotics. From the 185 strains of *S aureus* isolated, 42 strains were found to be resistant to oxacillin (MRSA), 09.02%, from the intensive care units 40,47%, surgery 28,57% and internal medicine and outpatients 21.42% and 9.5% respectively. Figure 1 shows the frequency of *S aureus* resistance to antibiotics, Figure 2 shows Agarose gel electrophores 1% of the multiplex PCR amplification products of the “mecA” and “nuc” genes, from S1 to S7 of the strains tested and Figure 3 shows agarose gel electrophores 1% of the multiplex PCR amplification products of the “mecA” and “nuc” genes, from S8 to S10 of the strains tested (SmartLader 200bp, Eurogentec).

## 4. Discussion

According to the results obtained, the number of staphylococci isolated from the pathological products are a bit high for males 58.06% compared to females 41.94%, with a sex ratio of 1.38. This result agrees well with that of (59.0%) found in a study at CHU Mostapha Bacha in Algiers by Antri et al.^[4]^ Sex is a risk factor for infection according to Oberholze.^[5]^ Most studies do not support this observation, apart from only 1 suggests that the rate of MRSA bacteremia would be higher in men than in women.^[6]^ Age is an important risk factor for patients, according to our results, the majority of patients who have contacted nosocomial infections are between 39 and 67 years old and a significant proportion at an age > 67 years. These results do not agree with the data that described age as an infectious risk factor beyond 1 year and after 75 years, or this can be explained by the high morbidity in the elderly.^[7]^

According to the graph it is found that there is a high resistance to *β*-lactams during years of study, 100% detect for penicillin followed by a high resistance to oxacillin 51.25% and cefoxitine 50%. This resistance is often associate with that of other classes of antibiotics such as Aminocidesamikacin/kanamycin 50%. These results are lower compared to the Tunisian study where resistance to these antibiotics was 78% amikacin/kanamycin.^[8]^ In Lebanon, the resistance of MRSA to aminoglycosides was represented by 2 relatively distant percentages; thus paradoxically at a low level (6.25%) with respect to gentamicin and tobramycin, reported a high percentage (90%) for amikacin and kanamycin.^[9]^

Macrolide resistance was: 28.29% Erythromycin and 07.31% Lincomycin is also high compared to that found in the United States and Tunisia erythromycin (66%) and (49%), respectively.^[8]^ Lincomycin is not an antibiotic widely used in Algeria for the treatment of MRSA infections, which is why we note that there is little resistance found compared to other countries. On the other hand, spiromycin and doxycilin remain among the most effective antibiotics against *S aureus* and more particularly MRSA with a percentage of 3.08% and 3.55%, respectively. Vancomycin is the most effective antibiotic on the strains studied.

The determination of the oxacillin-resistant character of the strains studied is based initially on the detection of the resistance of the strains by passing through the phenotypic tests such as antibiogram to see the resistance vis-à-vis cefoxitine and oxacillin then the confirmation of this resistance by molecular detection of genes responsible for antibiotic resistance (oxacillin) gene “mecA” by the use of PCR.

Multiplex PCR amplification using primers specific to the *S aureus* species “nuc” gene and primers responsible for oxacillin resistance gene “mecA” on the 1% agarose gel showed that all strains studied have the gene “nuc” which confirms their assignment to the genus *S.aureus*.

On the other hand, the resistance to oxacillin revealed 03 MRSA strain (S4, S9, S10) out of 10 tested layers (06 MRSA and 04 MSSA), this result expresses the presence of the mecA gene which is responsible for the resistance in the strains which the door these results are confirmed by the usual techniques of antibiogram.

In addition, the 03 mecA negative strains were confirmed to be MRSA by most of the phenotypic tests. This may be due to a point mutation or deletion of the mecA gene, or resistance to oxacillin by hyperproduction of β-lactamase so-called “Border line Oxacillin-Resistant *S aureus*” “BORSA,” these results consistent with that of Warren et al.^[10]^ Who found phenotypically resistant mecA negative isolates and who suggested that this resistance mediated by methods other than PLP2a.

Multi-resistant bacteria (BMR) present a significant challenge in healthcare settings, linked to the wide-ranging use of antibiotics. Our investigation focuses on the prevalence of *S aureus*, which demands specific attention as the isolated strains display resistance to β-lactam and other antibiotics like aminoglycosides, making therapeutic management challenging.

The rate of MRSA isolated in this study was 09.02%, similar to the rates observed in 2006 at Charlis Nicolle Hospital in Tunis which recorded a 10% rate of MRSA (Center for Coordination of Acting against Nosocomial Infections in the Paris-North Inter-Region, Central CLIN and Interclin Geriatric CLIN), Public Assistance Hospitals of Paris, 1998). It was also less important than the 32.7% obtained by Aouati et al^[11]^ at CHU Ben Badis of Constantine1.

The incidence of MRSA varies across different services. In the intensive care unit service, the rate of MRSA was found to be close to the signal study, at 40.47%, while external consultants showed a low incidence of 16.86%. These findings are consistent with previous studies indicating that hospitalization is the primary risk factor for MRSA acquisition. Since the late 1980s, research has demonstrated the transmission of MRSA to the community in patients who have not been hospitalized (also known as the carriage of MRSA).^[12]^ It is important to note that this finding is not based on subjective evaluations, and a logical structure has been maintained throughout with clear and concise language. The formal register has been maintained, while precision in word choice and grammatical correctness have been given due attention, resulting in a balanced and objective text.

The specific type of sample collected affects the obtained results. The highest prevalence was identified in pus samples at 42.13%, while Hemoculture represented the second most prevalent strain of MRSA at a rate of 26.61%. These findings show a slightly higher prevalence when compared to a similar study conducted in Morocco, which revealed a Hemoculture prevalence of 10.7% and a pus prevalence of 19%.

From an epidemiological point of view, the resistance by acquisition of the mecA gene was more serious because this gene was carried by a mobile genetic element that can not only promote the dissemination of resistance to methicillin but also carry other genes encoding the resistance to other antibiotics.^[12]^ Indeed *S aureus* has developed resistance to almost all the antibiotics on the market, among these main mechanisms of resistance was the synthesis of an enzyme activator, modification of the antibiotic target or the system of efflux. The main mechanism of resistance to methicillin was related to the modification of the target of beta-lactamine, by the synthesis of a fifth PLP (PLP2a) which has a low affinity to β-lactam.^[13]^

This PLP2a has the ability to polymerize the bacterial wall alone. The gene that codes for this PLP2a protein is called mecA.^[14]^ It is transported on a genetic element called Staphylococcal Chromosomal Cassete (SCC) mec (21–71kb) that integrates into a single site close to the origin *S.aureus* chromosome replication kit.^[3]^ The mec gene complex is a complex regulatory system with 2 repressor/anti-repressor systems of mecI/mecR1 and blaI/blaR.^[15,16]^ If the mecI/mecR1 system is functional, transcription of the mecA gene is strongly inhibited and the strain appears sensitive, with the usual antibiogram techniques, however, thanks to the blaI/blaR1 system, methicillin will slowly induce resistance.^[17]^

## 5. Conclusion

In Algeria the frequency of resistance of *S aureus* remains a little weak compared to other countries, but it continues to grow with time. Today, more than ever, the control of the spread of multidrug-resistant strains and the pressure generated by unjustified antibiotic prescriptions seems urgent, so it is necessary to be up to date to know the epidemiological profile of this germ and its susceptibility to antibiotics. To define at least 1 preventive control strategy and to control its development.

## Acknowledgments

We would like to thank everyone who contributed to the publication of this study, a big thank you to professor Allem (Bioresource Laboratory) for his moral support and also the staff of the institute pastor, daly brahim (Food Bacteriology laboratory) for the excellent provision of the laboratory and technic al assistance.

## Author contributions

**Conceptualization:** Fatima Zahra Dendi.

**Data curation:** Fatima Zahra Dendi.

**Formal analysis:** Fatima Zahra Dendi.

**Investigation:** Fatima Zahra Dendi.

**Methodology:** Fatima Zahra Dendi, Emmanuel Ifeanyi Obeagu.

**Supervision:** Mohammed Sebaihia.

**Validation:** Mohammed Sebaihia, Sidahmed Bensefia.

**Writing – original draft:** Fatima Zahra Dendi, Rachida Allem, Mohammed Sebaihia, Sidahmed Bensefia, Mohammed Cheurfa, Hannan Alamir, Emmanuel Ifeanyi Obeagu.

**Writing – review & editing:** Rachida Allem, Mohammed Sebaihia, Sidahmed Bensefia, Mohammed Cheurfa, Hannan Alamir, Emmanuel Ifeanyi Obeagu.
